# Physical Activity During Pregnancy and Gestational Weight Gain: Implications for Maternal–Fetal Epigenetic Programming and Long-Term Health

**DOI:** 10.3390/genes16101173

**Published:** 2025-10-06

**Authors:** Nektaria Zagorianakou, Stylianos Makrydimas, Efthalia Moustakli, Ioannis Mitrogiannis, Ermanno Vitale, George Makrydimas

**Affiliations:** 1 Department of Nursing, School of Health Sciences University of Ioannina, 4th Kilometer National Highway Str. Ioannina-Athens, 45500 Ioannina, Greece; zagorianakou@uoi.gr (N.Z.); ef.moustakli@uoi.gr (E.M.); 2 Medical School, Aristotle University of Thessaloniki, 54124 Thessaloniki, Greece; smakrydimas@gmail.com; 3 Harris Birthright Research Centre for Fetal Medicine, King’s College London, London SE5 8BB, UK; ioannismitrogiannisgr@gmail.com; 4 Department of Medicine and Surgery, University of Enna “Kore”, 94100 Enna, Italy; ermano.vitale@unikore.it; 5 Department of Obstetrics & Gynecology, University Hospital of Ioannina, 45500 Ioannina, Greece

**Keywords:** pregnancy, fetal development, epigenetics, DNA methylation, histone modification, offspring health, metabolic programming

## Abstract

**Background/Objectives**: Gestational weight gain (GWG) is a crucial factor influencing mother and fetal health, as high GWG is associated with adverse pregnancy outcomes and an increased long-term risk of obesity and metabolic issues in the children. In addition to controlling weight, maternal physical activity (PA) during pregnancy may influence fetal development through potential epigenetic mechanisms, including histone modifications, DNA methylation, and the production of non-coding RNA. **Methods**: This narrative review synthesizes evidence from randomized controlled trials (RCTs; *n* = 11, 3654 participants) investigating the impact of aerobic PA on GWG, while also highlighting emerging, primarily indirect findings on maternal–fetal epigenetic programming. **Results**: The majority of RCTs found that supervised PA interventions, especially when paired with nutritional counseling, decreased both the incidence of excessive GWG and total GWG. Enhancements in lipid metabolism, adipokine profiles, and maternal insulin sensitivity point to likely biochemical mechanisms that connect PA to epigenetic modification of fetal metabolic genes (e.g., IGF2, PGC-1α, LEP). Animal and observational studies suggest that maternal activity may influence offspring epigenetic pathways related to obesity and cardiometabolic conditions, although direct human evidence is limited. **Conclusions**: In addition to potentially changing gene–environment interactions throughout generations, prenatal PA is a low-cost, safe method of improving maternal and newborn health. Future RCTs ought to incorporate molecular endpoints to elucidate the epigenetic processes by which maternal exercise may provide long-term health benefits.

## 1. Introduction

Pregnancy is a complex physiological state requiring comprehensive clinical monitoring to ensure optimal maternal and fetal outcome. Standard prenatal care includes screening for chromosomal aneuploidies [1], assessment of fetal anatomy and growth [2], and evaluation of maternal risk for complications such as gestational diabetes mellitus (GDM) and preeclampsia [3]. Gestational weight gain (GWG) is an important clinical parameter, as deviations from established recommendations are associated with increased maternal and neonatal morbidity [4,5]. Accordingly, at each routine prenatal visit, healthcare providers should assess maternal weight and provide individualized, evidence-based guidance to support gestational weight gain within the ACOG-recommended ranges, which are approximately 12.5–18 kg for underweight, 11.5–16 kg for normal weight, 7–11.5 kg for overweight, and 5–9 kg for obese women [6].

Excessive GWG (EGWG) is a significant public health concern, associated with preterm birth, cesarean delivery, fetal macrosomia, infant mortality, GDM, and hypertensive disorders of pregnancy [7,8]. Additionally, EGWG has been implicated in adverse long-term outcomes for offspring through mechanisms of metabolic programming, increasing susceptibility to obesity, insulin resistance, and type 2 diabetes [9,10]. Both low and high birthweights independently heightened risks for obesity and cardiovascular disease in later life, underscoring the intergenerational impact of maternal weight management [11,12,13]. Globally, clinicians advocate for personalized lifestyle interventions to support appropriate GWG.

Physical activity (PA) during pregnancy has been consistently associated with improvements in physical and mental health [14,15], and a growing body of evidence suggests its potential in reducing risks of adverse outcomes including preterm birth and delivery complications [16,17,18,19,20,21,22]. Adherence to international guidelines, such as those from the World Health Organization, facilitates the safe integration of PA into prenatal care [23]. By improving placental perfusion, enhancing insulin sensitivity, and modulating inflammatory cytokines, maternal PA optimizes the intrauterine environment. Experimental and observational research also indicates that exercise may influence epigenetic modifications, such as DNA methylation and histone changes in genes that control growth and energy metabolism (e.g., *IGF2, LEP, PGC-1α*), which could reduce the risk of metabolic disease in offspring. However, direct evidence from human randomized controlledtrials (RCTs) remains limited, and results are heterogeneous depending on PA type, intensity, and adherence.

The framework for comprehending how maternal environment and behaviors, like exercise, may leave molecular “signatures” that endure across generations has been made possible by recent developments in epigenetics [24]. This notion is in line with the Developmental Origins of Health and illness (DOHaD) hypothesis, which holds that exposure to certain environmental factors during crucial developmental windows can influence a person’s chance of developing an illness in later life [25]. Maternal PA, for instance, has been linked to modifications in the methylation patterns of growth-related and metabolic genes (such as *IGF2, LEP, and PGC-1α*), indicating that lifestyle modifications may impact the regulation of fetal genes [26,27]. These results demonstrate PA’s potential as a gene-environment interaction modulator of intergenerational health in addition to its use as a GWG management method [28].

Many high-income nations estimate that 40–60% of people have EGWG, and rates are said to be rising worldwide. This rising trend highlights the pressing need for efficient preventative measures and coincides with rising rates of maternal obesity [29]. Significant gaps still exist despite the evidence supporting PA, including the optimal type, level, and timing of exercise during pregnancy, as well as the application of molecular results from animal models to humans. It is imperative to fill these gaps to improve maternal–fetal health outcomes globally and inform clinical recommendations [30].

This narrative review synthesizes current evidence from randomized controlled trials on the relationship between aerobic PA and GWG. It further explores potential molecular and epigenetic mechanisms, emphasizing that most human evidence is indirect. The aim is to provide obstetricians, gynecologists, and researchers with a comprehensive, evidence-based understanding of PA in pregnancy management and its possible implications for intergenerational health.

## 2. Methods

### 2.1. Literature Search and Study Selection

A narrative review of the literature was conducted to explore the association between PA, particularly aerobic exercise, and GWG in pregnant women. PubMed and Scopus databases were searched for articles published in English from January 2000 to June 2024. Search terms included combinations of “gestational weight gain” AND “pregnancy,” “excessive gestational weight gain” AND “physical activity,” and “excessive gestational weight gain” AND “exercise”. We acknowledge that alternative terminology such as “maternal exercise,” “prenatal physical activity,” or “weight control in pregnancy” may have identified additional studies.

**Figure 1:**
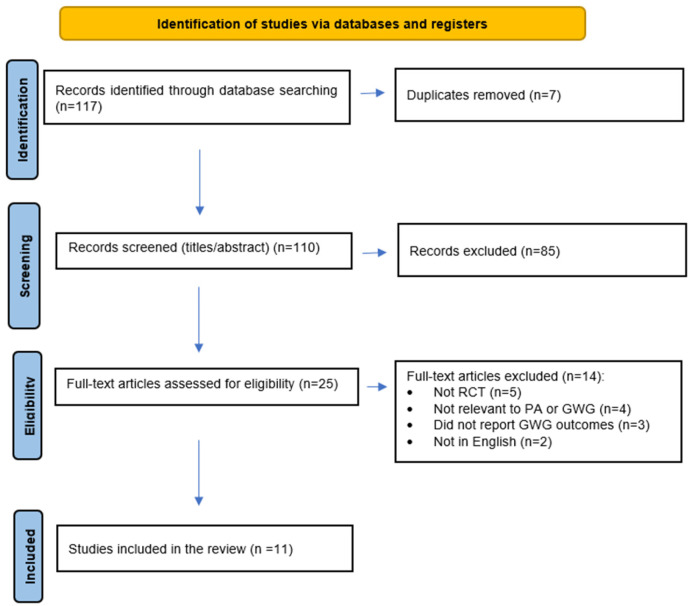
PRISMA flow diagram illustrating the study selection process. Abbreviations: RCT = Randomized Control Trial; GWG = gestational weight gain; PA = physical activity. Note: NS indicates results that were not statistically significant.

The search initially identified 117 articles. After title and abstract screening, studies were excluded for irrelevance, duplicate publication, or failure to meet eligibility criteria. Screening and data extraction were performed independently by two authors, and discrepancies were resolved by discussion. Full-text review was then performed, and 11 articles were retained for final synthesis. The detailed selection process is presented in Figure 1.

### 2.2. Inclusion Criteria

Studies were included if they met the following criteria: (1) GWG was reported as a primary or secondary outcome; (2) the population consisted of pregnant women, including those who conceived via in vitro fertilization (IVF); and (3) the intervention involved aerobic exercise exclusively. Studies were considered regardless of whether the reported association between PA and GWG was positive, negative, or neutral.

### 2.3. Exclusion Criteria

Studies were excluded if they met any of the following conditions: (1) study design was a case report, case series, meta-analysis, systematic review, umbrella review, or study protocol; (2) the population involved non-human species, non-pregnant or postpartum individuals; (3) the focus was on genes, proteomics, or epigenetic changes rather than PA and GWG; (4) the study was retracted or not published in English; or (5) the primary topic was unrelated, such as COVID-19. Systematic reviews and meta-analyses were intentionally excluded to avoid duplication of data and maintain the focus on primary RCT evidence. A quality assessment was performed for all included studies using Cochrane Risk of Bias Tool (RoB 2.0). The risk of bias was evaluated in each study according to the following criteria: low risk, moderate concerns, or high risk. These criteria included the randomization method, variations from intended interventions, missing outcome data, measurement of outcomes, and selection of the reported results. The majority of studies demonstrated a low risk of bias for outcome reporting and randomization; however, certain issues with missing outcome data and blinding of outcome assessment were noted in a few trials.

## 3. Results

Following the application of the inclusion and exclusion criteria, the final review contained 11 RCTs with a total of 3654 participants. These studies assessed how GWG and associated maternal and newborn outcomes were affected by supervised aerobic exercise regimens, either with or without dietary advice.

The majority of RCTs demonstrated that PA treatments improved overall GWG, especially when combined with dietary counseling and when adherence was high. Several studies reported significant reductions in total GWG, incidence of excessive GWG, or improved metabolic parameters such as insulin sensitivity and neonatal adiposity. A detailed summary of the included studies is presented in Table 1.

### 3.1. Effects of Physical Activity on Gestational Weight Gain

Among the RCTs reviewed, several studies reported favorable effects of PA interventions on GWG. Ruchat et al. [40] found that women assigned to an exercise and diet regimen had over a 70% likelihood of remaining within the Institute of Medicine (IOM) recommendations (χ^2^ = 4.72, *p* = 0.32, not significant). Wang et al. [35] observed significant differences in GWG between intervention and control groups both at mid-pregnancy (4.08 ± 3.02 vs. 5.92 ± 2.58 kg; *p* < 0.001) and at term (8.38 ± 3.65 vs. 10.47 ± 3.33 kg; *p* < 0.001). Similarly, Nobles et al. [36] reported a 30% reduction in the odds of exceeding GWG guidelines (OR = 0.69, *p* = 0.30, not significant), though the 1 kg difference between groups was not statistically significant.

Other studies also demonstrated benefits of exercise programs. Barakat et al. (2018) [32] reported lower total GWG (12.19 vs. 13.33 kg; *p* = 0.005) and reduced incidence of EGWG (30.2% vs. 20.5%; OR = 0.597; *p* = 0.018) in the exercise group. Pelaez et al. [31] observed that participants with BMI ≥ 25 kg/m^2^ were less likely to exceed GWG recommendations (22.0% vs. 34.3%; *p* = 0.03). Barakat et al. (2014) [38] similarly found EGWG significantly less common among women engaging in exercise programs (21.2% vs. 35.6%; *p* = 0.02). The BHIP study suggested a trend toward meeting IOM criteria when combining diet and PA, although differences did not reach statistical significance [42].

In contrast, several trials reported no significant differences in GWG. McDonald et al. [41] observed slightly higher GWG in the intervention group (29.0 vs. 25.0 kg; *p* = 0.22). Nitert et al. [39] found no differences among obese participants (7.87 ± 4.00 vs. 8.28 ± 6.10 kg; *p* = 0.81), attributing this to low adherence. da Silva et al. [34], the PAMELA study [33], and Brik et al. [33] also reported non-significant GWG differences, although Brik et al. noted improvements in fetal cardiac function and postpartum weight recovery.

Most RCTs indicated that PA, particularly when combined with dietary advice and implemented with sufficient rigor and adherence, can help control gestational weight gain, although results are somewhat mixed and epigenetic effects have not been directly assessed [43,44].

### 3.2. Maternal Metabolic and Physical Health Outcomes

Beyond GWG, PA interventions demonstrated benefits for maternal metabolic health. Wang et al. [35] reported improvements in insulin sensitivity and reductions in neonatal birth weight among participants engaging in structured aerobic exercise. The DALI study by Simmons et al. [37] showed that combining healthy eating with PA reduced GWG in obese women, although metabolic outcomes were only modestly improved. Pelaez et al. [31] additionally noted enhanced maternal self-perception and physical fitness, highlighting the multidimensional benefits of prenatal exercise.

### 3.3. Fetal and Neonatal Outcomes

Potential benefits for the fetus were noted in several trials. Exercise groups exhibited improved fetal cardiac function, improved birth weight distribution, and decreased neonatal adiposity, indicating that maternal PA may have a favorable effect on the intrauterine environment. These metabolic and developmental advantages suggest that PA may have independent health benefits for offspring, even in cases where GWG was not considerably changed.

### 3.4. Epigenetic and Molecular Mechanisms

**Figure 2:**
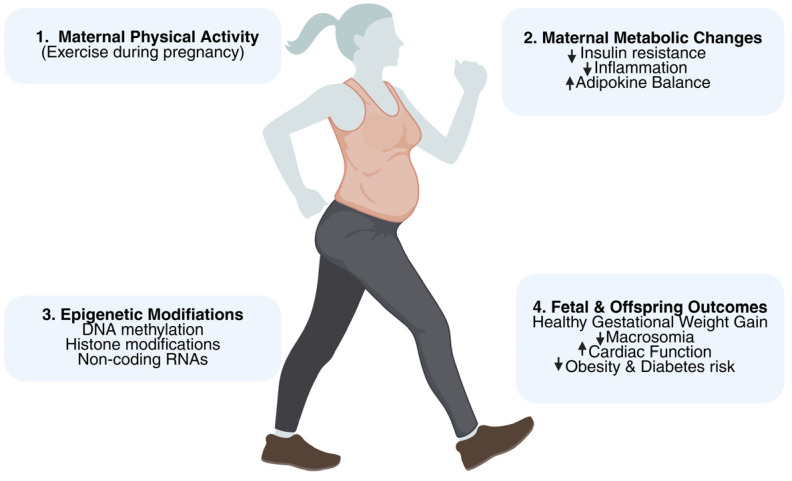
Pathway from exercise to maternal physiology, then epigenetic changes, and finally to intergenerational health benefits. These steps are based on limited and indirect human data and should not be interpreted as established causal effects. ↓ = low; ↑ = high.

Maternal PA may alter development and metabolism-related molecular pathways, according to observational and animal research, even though none of the RCTs specifically evaluated epigenetic effects. Among the possible processes are changes in DNA methylation, histone modifications, and the expression of non-coding RNA in the placenta and cord blood, namely in genes like *IGF2, LEP*, and *PGC-1α* [45,46]. These molecular changes may partly explain how prenatal exercise contributes to long-term offspring health and support the concept of intergenerational benefits of maternal PA [47] (Figure 2).

## 4. Discussion

### 4.1. Impact of PA on GWG and Maternal Outcomes

This narrative review highlights the complex yet promising role of PA during pregnancy in regulating GWG and promoting maternal–fetal health. The majority of reviewed studies support the beneficial impact of structured exercise programs in helping women remain within the IOM guidelines for GWG, particularly when PA is combined with dietary counseling and sustained adherence [31,32,36,40,43].

Several trials reported significant reductions in total GWG and the incidence of EGWG. Notably, Barakat et al. and Wang et al. demonstrated potential improvements in maternal weight control and metabolic parameters, including enhanced insulin sensitivity and reduced neonatal birth weight [32,35]. These findings are clinically important as they suggest that PA may mitigate complications such as gestational diabetes mellitus (GDM), hypertensive disorders, and cesarean delivery while supporting normal birth outcomes and faster postpartum recovery [30,33].

### 4.2. Variability Among Studies and Role of Adherence

Not all studies observed consistent effects. McDonald et al. and Nitert et al. found no significant differences in GWG between intervention and control groups [39,41]. Such discrepancies may be attributed to variations in study design, participant characteristics (e.g., baseline BMI), type and intensity of PA interventions, and adherence rates. In particular, trials involving obese women often reported low compliance with exercise protocols, emphasizing that adherence is a critical determinant of intervention success [39,40]. A recurrent problem was low adherence, especially among obese women, indicating that compliance is a critical factor in determining the effectiveness of interventions. To increase participation and adherence, future research could use tactics like behavioral support, supervised sessions, or digital engagement tools.

### 4.3. Broader Maternal and Fetal Benefits

Beyond weight management, PA during pregnancy offers additional maternal benefits, including improved mood, reduced postpartum depression risk, better cardiovascular health, and reduced postpartum weight retention [31,36,40]. Enhanced maternal self-perception and physical fitness have also been reported [31,36,39,43]. From a fetal perspective, regular maternal exercise appears safe and may even confer advantages such as improved cardiac function and a reduced risk of macrosomia, a factor often associated with delivery complications [33].

### 4.4. Practical Recommendations for Clinicians

Clinicians should encourage pregnant women without contraindications to participate in at least 150 min of moderate-intensity aerobic exercise per week, preferably in 30- to 60-min sessions three to five times a week, according to current research and international guidelines [14,23]. The probability of reaching the recommended GWG is increased when PA is combined with customized nutritional counseling. To guarantee safety, exercise routines should be tailored to the woman’s fitness level and trimester, avoiding contact sports and supine positions after mid-pregnancy.

### 4.5. Barriers and Equity Considerations

Participation in PA may be restricted by cultural or financial barriers, lack of time, pregnancy-related exhaustion, and access to safe exercise facilities. Reducing disparities and enhancing adherence may be achieved by addressing these barriers through group classes, home-based programs, culturally specific education, and integration into regular prenatal visits. Many studies differ in intervention protocols, participant demographics, and outcome measures, limiting the generalizability of results [48], and few trials have explored the potential molecular or epigenetic mechanisms underlying the observed effects [49].

### 4.6. Epigenetic and Intergenerational Implications

Emerging evidence, primarily from animal models and observational studies, suggests that maternal PA may influence fetal epigenetic programming by modulating DNA methylation and histone modifications in metabolic pathways, potentially impacting offspring health in the long term. However, this remains an underexplored area requiring rigorous investigation [50].

From the standpoint of genes and environments, PA might act as a molecular regulator of intrauterine development. The metabolic environment resulting from exercise-induced improvements in maternal glucose regulation, adipokine secretion, and inflammatory balance can alter epigenetic markers in fetal tissues placenta [51]. Intergenerational programming effects are plausible, as evidenced by research conducted outside of the RCT paradigm that have connected maternal PA with altered DNA methylation in genes associated with growth and metabolism, including *IGF2*, *LEP*, and *PGC-1α* [50]. Similarly, non-coding RNA regulation and histone modifications might act as additional epigenetic control layers that enable maternal behavior to influence the phenotypes of her offspring [52].

### 4.7. Future Research and Directions

In addition to objectively tracking adherence, future RCTs should develop standardized exercise regimens tailored to diverse maternal demographics. To clarify underlying mechanisms, studies should include molecular endpoints such as placental omics analyses, longitudinal evaluations of cord blood DNA methylation, histone modifications, and non-coding RNA profiles. Understanding how maternal activity affects the health trajectories of subsequent generations would be advanced by integrating these methods with clinical outcomes like prenatal weight gain and child metabolic health.

### 4.8. Novelty and Contribution

To the best of our knowledge, this review is the first attempt to combine mechanistic theories about the epigenetic and intergenerational impacts of maternal physical activity with data from RCTs on GWG. This work offers a distinctive viewpoint that connects clinical outcomes and possible underlying biological pathways by fusing solid clinical trial data with new molecular discoveries. Emphasizing this integration highlights the manuscript’s value addition for clinicians and researchers interested in maternal–fetal health, and it may help guide the planning of further translational studies.

## 5. Conclusions

Optimizing GWG and reducing the risk of excessive weight gain during pregnancy can be achieved safely and effectively through PA. Evidence from randomized controlled trials supports its role in enhancing maternal metabolic health, decreasing pregnancy complications, and promoting positive neonatal outcomes. PA therapies are most effective when combined with nutritional counseling and consistent adherence. Incorporating structured exercise programs into routine prenatal care can help mothers and their unborn children experience healthier pregnancies and improved long-term health.

## Figures and Tables

**Table 1:** Summary of included RCTs on aerobic PA during pregnancy outcomes related to GWG.

First Author (Year)	Country	Sample Size	Intervention (Frequency/Timing)	Population Characteristics	Primary/Secondary Outcomes	Key Findings
Pelaez et al. (2019) [31]	Spain	345	Moderate–vigorous exercise 3×/week from 12–38 weeks	BMI stratified; avg age ~30	GWG, adherence, GDM	↓EGWG (esp. BMI ≥ 25), improved fitness
Barakat et al. (2019) [32]	Spain	594	Moderate exercise 3×/week 8–39 weeks	Healthy singleton pregnancies	GWG, GDM incidence	↓Total GWG, ↓GDM
Brik et al. (2019) [33]	Spain	120	60-min sessions 3×/week	Low-risk pregnancies	GWG, fetal cardiac function	No GWG reduction, improved fetal cardiac parameters
da Silva et al. (2017) [34]	Brazil	639	Exercise 3×/week 16–36 weeks	Mixed-risk population	GWG, preterm birth, GDM	NS
Wang et al. (2017) [35]	China	300	Cycling 3×/week < 13–37 weeks	Overweight/obese women	GWG, GDM, insulin resistance	↓GWG, ↓GDM, ↓birth weight
Nobles et al. (2018) [36]	USA	241	12-week structured program	Ethnically diverse	GWG (primary), adherence	30% ↓odds EGWG (NS)
Simmons et al. (2016) [37]	Europe	436	HE, PA, HE + PA interventions	Obese women	GDM risk, GWG	HE + PA ↓GWG; metabolic outcomes NS
Barakat et al. (2012) [38]	Spain	200	Moderate exercise entire pregnancy	Healthy nulliparas	GWG, maternal/fetal outcomes	↓EGWG, safe intervention
Dekker Nitert et al. (2015) [39]	Australia	35	Individualized plan	Obese women	GWG, metabolic markers	NS
Ruchat et al. (2012) [40]	Canada	94	Walking + nutrition control	Normal BMI women	GWG, postpartum weight	↑IOM compliance, ↓postpartum weight
McDonald et al. (2021) [41]	USA	128	Moderate exercise < 16 wks–delivery	Healthy pregnancies	Neonatal morphometry, GWG	↓neonatal adiposity; GWG slightly ↑ (NS)

Abbreviations: GWG = gestational weight gain; EGWG = excessive gestational weight gain; GDM = gestational diabetes mellitus; HE = healthy eating; NS = not significant; PA = physical activity; IOM = Institute of Medicine. Note: NS indicates results that were not statistically significant. ↓ = low; ↑ = high.

## Data Availability

No new data were created or analyzed in this study.

## Abbreviations

The following abbreviations are used in this manuscript:

GWG	Gestational Weight Gain
PA	Physical Activity
RCTs	Randomized Control Trials
EGWG	Excessive Gestational Weight Gain
DOHaD	Developmental Origins of Health and Illness
